# Sole Infrequent Karyotypic Aberration Trisomy 6 in a Patient with Acute Myeloid Leukemia and Breast Cancer in Remission

**DOI:** 10.4274/tjh.2016.0030

**Published:** 2017-03-01

**Authors:** Mürüvvet Seda Aydın, Süreyya Bozkurt, Gürsel Güneş, Ümit Yavuz Malkan, Tuncay Aslan, Sezgin Etgül, Yahya Büyükaşık, İbrahim Celalettin Haznedaroğlu, Nilgün Sayınalp, Hakan Göker, Haluk Demiroğlu, Osman İlhami Özcebe, Salih Aksu

**Affiliations:** 1 Hacettepe University Faculty of Medicine, Department of Adult Hematology, Ankara, Turkey; 2 Hacettepe University Cancer Institute, Basic Oncology, Ankara, Turkey

**Keywords:** Trisomy 6, Acute myeloid leukemia, Breast cancer

## TO THE EDITOR,

**Figure 1:**
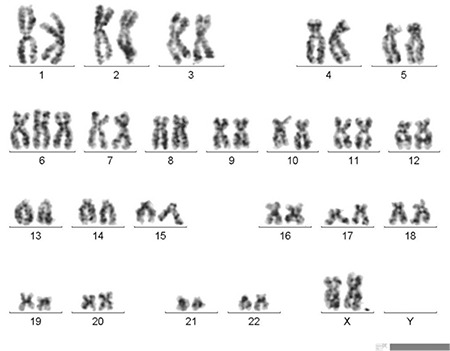
Bone marrow cytogenetic analysis revealed trisomy 6 (47,XX, +6 [20]) in all the metaphases.

Cytogenetic abnormalities play important roles in the diagnosis and prognosis of leukemias [1]. Trisomy 6 as the sole karyotypic aberration is infrequent in leukemias [1,2]. A 50-year-old female patient presented with fatigue. She had been treated by mastectomy and given chemotherapy (no further information available) for breast cancer 3 years ago. She had been using tamoxifen for 3 years. Her breast cancer was in remission. Physical examination was consistent with a pale appearance. Hemoglobin, neutrophils, and platelet count were 8.5 g/dL, 900/µL, and 11,000/µL, respectively, on admission. In the peripheral blood smear, there were dysplastic features in monocytes and a few blasts were reported. In flow cytometry, CD13, CD33, CD34, CD45, CD117 (c-kit), HLA-DR, and MPO were positive. Bone marrow aspiration and biopsy revealed hypercellularity with dysplastic and megaloblastic features in erythroid series, grade 1/3 reticulin fibrosis, and 24% blasts without ring sideroblasts, which in turn with cytometry findings were accepted as evidence of acute myeloid leukemia (AML). Bone marrow cytogenetic analysis revealed trisomy 6 (47,XX, +6 [20]) in all the metaphases (Figure 1). The patient was not in remission after the first induction treatment and she passed away due to septic shock during the second induction treatment.

Chromosome aberrations detected in therapy-related AML (t-AML) and de novo AML cases are identical but their frequencies may differ [3]. In a series at the University of Chicago, normal karyotypes were seen in 9.6% of t-AML cases [4]. In the report of Godley and Larson, among 306 patients with t-AML, 32 had solid breast cancer as the primary diagnosis [5]. Alkylating exposures and topoisomerase II inhibitors are associated with t-AML [3,6]. Godley and Larson mentioned granulocyte colony-stimulating factor usage as a risk factor in t-AML after breast cancer [5]. Unfortunately, we do not know which agents were given for our patient’s breast cancer.

Autosomal trisomies have been described in several hematologic malignancy cases. The first case of sole trisomy 6 was reported in aplastic anemia. Other reports showed that trisomy 6 was associated with hypoplastic bone marrow, dyserythropoiesis, and AML [7]. Mohamed et al. reviewed 7 patients with trisomy 6. Patients presenting with overt AML had hyperplastic marrows [8]. Our patient had hypercellular marrow, as well. Mohamed et al. also reviewed the literature and found 4 MDS cases among 22 patients with trisomy 6 [8]. The marrow examination of this case revealed secondary dysplastic leukemia. The patient of Gupta et al. had de novo AML and did not respond to the first remission induction treatment [1].

Yu et al. reviewed ten reports in PubMed describing 18 cases of AML presenting with trisomy 6 as the sole karyotypic abnormality along with 3 cases of their own [7]. They concluded that there were no direct correlations between the number of blasts and the percentage of abnormal metaphases. They could not identify any correlation between morphology or prognosis and trisomy 6 [7].

Under these circumstances, as in our case, we lack information on the impact of trisomy 6 on prognosis in secondary AML patients.

## References

[1] Gupta M, Radhakrishnan N, Mahapatra M, Saxena R (2015). Trisomy chromosome 6 as a sole cytogenetic abnormality in acute myeloid leukemia. Turk J Hematol.

[2] Choi J, Song J, Kim SJ, Choi JR, Kim SJ, Min YH, Park TS, Cho SY, Kim MJ (2010). Prognostic significance of trisomy 6 in an adult acute myeloid leukemia with t(8;21). Cancer Genet Cytogenet.

[3] Pedersen-Bjergaard J, Andersen MT, Andersen MK (2007). Genetic pathways in the pathogenesis of therapy-related myelodysplasia and acute myeloid leukemia. Hematology Am Soc Hematol Educ Program.

[4] Qian Z, Joslin JM, Tennant TR, Reshmi SC, Young DJ, Stoddart A, Larson RA, Le Beau MM (2010). Cytogenetic and genetic pathways in therapy-related acute myeloid leukemia. Chem Biol Interact.

[5] Godley LA, Larson RA (2008). Therapy-related myeloid leukemia. Semin Oncol.

[6] Zhang L, Wang SA (2014). A focused review of hematopoietic neoplasms occurring in the therapy-related setting. Int J Clin Exp Pathol.

[7] Yu S, Kwon MJ, Lee ST, Woo HY, Park H, Kim SH (2014). Analysis of acute myeloid leukemia in Korean patients with sole trisomy 6. Ann Lab Med.

[8] Mohamed AN, Varterasian ML, Dobin SM, McConnell TS, Wolman SR, Rankin C, Willman CL, Head DR, Slovak ML (1998). Trisomy 6 as a primary karyotypic aberration in hematologic disorders. Cancer Genet Cytogenet.

